# Fe–Decorated Nitrogen–Doped Carbon Nanospheres as an Electrochemical Sensing Platform for the Detection of Acetaminophen

**DOI:** 10.3390/molecules28073006

**Published:** 2023-03-28

**Authors:** Xiangchuan Zhao, Liping Zhang, Zhaoyun Chu, Qing Wang, Yue Cao, Jun Cao, Jiao Li, Wu Lei, Boming Zhang, Weimeng Si

**Affiliations:** 1 School of Materials Science and Engineering, Shandong University of Technology, Xincunxi Road 266th, Zibo 255000, China; 2 Institute of Advanced Materials, Shandong Institutes of Industrial Technology, Jinan 250000, China; 3 School of Chemistry and Chemical Engineering, Nanjing University of Science and Technology, Xiaolingwei 200th, Nanjing 210094, China

**Keywords:** electrochemical sensor, melamine–formaldehyde resin, acetaminophen, Fe–decorated nitrogen–doped carbon nanospheres

## Abstract

In this work, Fe–decorated nitrogen–doped carbon nanospheres are prepared for electrochemical monitoring of acetaminophen. Via a direct pyrolysis of the melamine–formaldehyde resin spheres, the well–distributed Fe–NC spheres were obtained. The as–prepared Fe–NC possesses enhanced catalysis towards the redox of acetaminophen for abundant active sites and high–speed charge transfer. The effect of loading Fe species on the electrochemical sensing of acetaminophen is investigated in detail. The synergistic effect of nitrogen doping along with the above–mentioned properties is taken advantage of in the fabrication of electrochemical sensors for the acetaminophen determination. Based on the calibration plot, the limits of detection (LOD) were calculated to be 0.026 μM with a linear range from 0–100 μM. Additionally satisfactory repeatability, stability, and selectivity are obtained.

## 1. Introduction

Acetaminophen (AC), also known as paracetamol, is clinically used to relieve fever, colds, and a variety of pain, such as headache, and toothache, which is an alternative to aspirin [1,2]. However, using AC in excess of the prescribed dose can cause side effects. Therefore, it is essential to develop a sensitive, quick, and easy AC concentration detection method. Currently, many methods have been developed for the detection of AC, such as spectrophotometry [3], high–performance liquid chromatography [4], chemiluminescence [5], fluorescence spectroscopy [6], capillary electrophoresis [7], etc. However, the above methods usually require complex preprocessing, which is time–consuming, expensive, and requires professionals, so it is difficult to achieve large–scale applications [8]. The electrochemical approach has the benefits of being affordable, portable, highly sensitive, and exceptionally stable, as well as having excellent reproducibility and a quick turnaround time for analysis [9,10]. Therefore, the electrochemical detection method of paracetamol has been attracting increasing attention in recent years [11,12].

Carbon materials have the advantages of rich content, low price, and good chemical stability, which are subject to many research fields [13,14]. However, its catalytic activity has difficulty meeting the needs of sensors. The modification of carbon materials can endow them with new physical and chemical properties and improve their catalytic ability. Therefore, in recent years, transition metal and nitrogen co–doped carbon materials (M–NC) have attracted wide attention. Among them, Fe–NC materials are favored by researchers due to iron reserves, low prices, and low biological toxicity. As far as we know, the study of Fe–NC materials as electrochemical sensors for detecting AC is limited. In addition, there are some reports that Fe/Fe_3_C nanoparticles can provide more electrons for the active sites of Fe–Nx to enhance its catalytic activity [15,16,17]. Inspired by this, we hope to prepare a Fe–N–C material containing both Fe and Fe_3_C for the detection of AC.

Because raw materials are cheap, melamine–formaldehyde (MF) resin is considered to be the dominant precursor for the synthesis of nitrogen–doped carbon materials compared to other polymers such as polydopamine and polypyrrole with higher nitrogen content and rich amino groups [18]. In the pyrolysis process, a large number of triazines in MF can be stabilized in the polymer skeleton to reduce the loss of nitrogen. Herein, we report a method to synthesize Fe–NC materials using MF resin as the carbon and nitrogen sources and ferric chloride as the iron source. Using nitrogen in MF resin as the anchor point for iron atoms, a large number of triazine groups in MF can provide abundant nitrogen through pyrolysis to form Fe–Nx. Meanwhile, graphitic carbon–encapsulated Fe–/Fe_3_C nanoparticles are formed, which helps to enhance the stability of the material. We demonstrated the formation of Fe–Nx active sites and Fe/Fe_3_C nanoparticles by a series of characterization methods, and the composite modified electrode was successfully used for electrochemical detection of AC.

## 2. Results and Discussion

### 2.1. Structure and Morphology Studies

**Figure 1:**
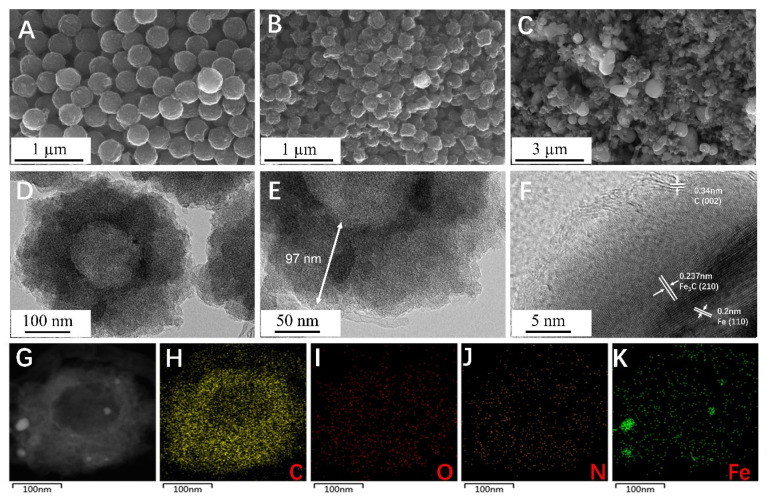
SEM images of Fe–NC–700 (**A**), Fe–NC–800 (**B**), Fe–NC–900 (**C**); TEM images of Fe–NC–800 (**D**–**F**), the arrow in (**E**) represents the thickness of the graphite carbon layer of the carbon sphere; EDS mapping images of Fe–NC–800 (**G**–**K**).

Firstly, melamine and formaldehyde were polymerized under a low temperature water bath to produce an MF resin prepolymer, then ferric chloride was added for further polymerization to obtain an Fe–doped MF resin, and PVA was used as dispersant to prevent MF resin aggregation. The as–synthesized MF resin (Figure S1A) was spherical with a rough surface and a diameter of approximately 700 nm. The morphology after carbonization is shown in Figure 1. The spherical structure of the MF resin is retained, but the diameter is significantly reduced. It indicates that the volume of the MF resin shrinks during the pyrolysis process, and the shrinkage rate can be up to 50%. With NC–800 (Figure S1B) and Fe–NC–800 (Figure 1B), the doping of Fe makes the surface of the samples rougher. This rough surface helps to increase the specific surface area of the material and increases the contact area between the material and the substance to be tested. By comparing the morphologies of Fe–NC–700 (Figure 1A), Fe–NC–800 (Figure 1B), and Fe–NC–900 (Figure 1C), it can be seen that the diameter gradually increases, and the spherical shape becomes more and more irregular with the increase in the pyrolysis temperature, until the pyrolysis temperature reached 900 °C and the spherical structure was destroyed.

TEM images (Figure 1D–F) show the microstructure of Fe–NC–800; it is a carbon sphere with a diameter of approximately 300 nm, with nanoparticles dispersed or embedded on the surface or inside of the carbon sphere. The thickness of the carbon layer is approximately 97 nm. HRTEM shows that the lattice spacing of 0.2 nm and 0.237 nm correspond to the (110) crystal plane of Fe and the (210) crystal plane of Fe_3_C, respectively [19]. The (002) crystal plane of graphite carbon corresponds to a lattice spacing of 0.34 nm [20]. This indicates that the nanoparticles are composed of Fe and Fe_3_C, and the structure of the nanoparticles wrapped by the graphite carbon layer can not only avoid contact between the active center and the reaction medium, but also improve the stability of the material. Additionally, the wrapped nanoparticles can encourage the transport of electrons from Fe_3_C to the outermost layer of graphite carbon, increasing the material’s catalytic activity [21]. The successful doping of Fe is demonstrated by the uniform distribution of C, N, O, and Fe elements on the mapped image surface in Figure 1G–K.

**Figure 2:**
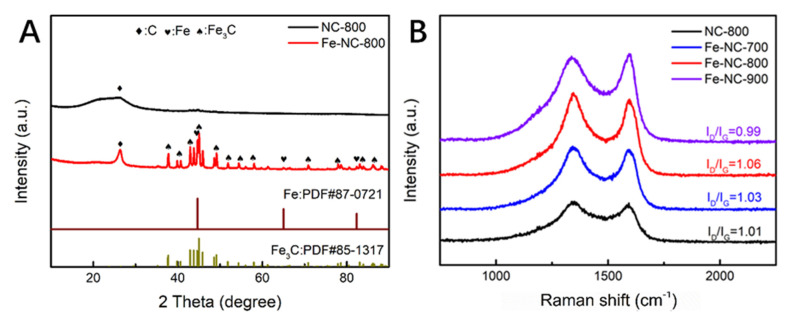
(**A**) the XRD of NC–800, Fe–NC–800; (**B**) the Raman spectrum of NC–800, Fe–NC–700, Fe–NC–800, Fe–NC–900.

By using XRD, the material’s crystal phase was examined (Figure 2A). The graphite carbon (002) crystal plane is indicated from diffraction peaks on NC–800 and Fe–NC–800 at 26.4° (PDF#26–1080). The fact that Fe can accelerate the graphitization of carbon is demonstrated by the fact that the diffraction peak intensity of Fe–NC–800 is higher than that of NC–800 and the peak width is narrower [22]. The lattice spacing in HRTEM linking with the diffraction peaks at 37.8° and 44.6°, which is assigned to the (210) plane of Fe_3_C and (110) plane of Fe, respectively (PDF#85–1317, PDF#87–0721), demonstrates that the nanoparticles are made of Fe and Fe_3_C.

Figure 2B depicts the Raman spectra of NC–800, Fe–NC–700, Fe–NC–800, and Fe–NC–900. At 1351 cm^−1^ and 1582 cm^−1^, respectively, typical disordered carbon (*D*–band) and graphite carbon (*G*–band) structures were seen. The degree of graphitization of carbon materials can be determined by the intensity ratio of *I_D_*/*I_G_* [22]. The *I_D_*/*I_G_* value of Fe–NC–800 (1.06) is higher than that of NC–800 (1.01) at the same pyrolysis temperature, demonstrating that Fe–doping causes carbon materials to have higher defect levels. Fe–NC–800 has the highest *I_D_*/*I_G_* value because the surface of spherical particles becomes rougher as the pyrolysis temperature increases from 700 °C to 800 °C, increasing specific surface area and strengthening the *D* band. When the temperature rises from 800 °C to 900 °C, the spherical structure is destroyed, and the volatilization of nitrogen at high temperature leads to the decrease in defect degree [23].

**Figure 3:**
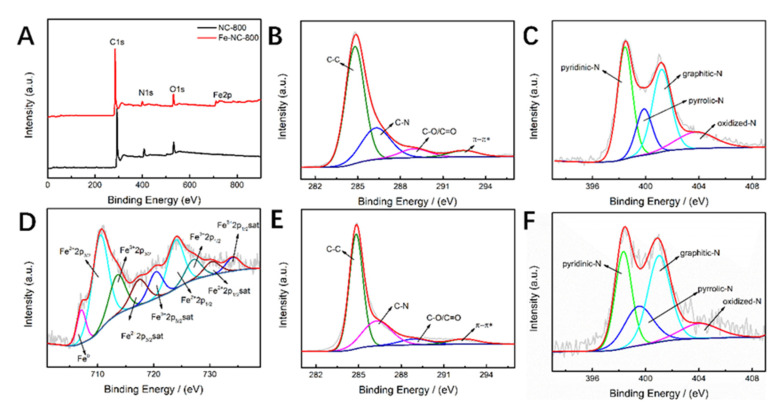
XPS survey spectra of prepared samples (**A**); C1s spectra of NC–800 (**B**) and Fe–NC–800 (**E**); N1s spectra of NC–800 (**C**) and Fe–NC–800 (**F**); Fe2p spectrum of Fe–NC–800 (**D**), in (**B**,**D**), * represents bonding π electron transitions to π* orbitals.

By using X–ray photoelectron spectroscopy (XPS), the elemental composition of the NC–800 and Fe–NC–800 were examined (Figure 3A), demonstrating the successful incorporation of Fe. Fe–NC–800 has the following atomic percentages: C (88.61%), O (5.33%), N (4.02%), and Fe (2.04%). The C–C (284.8 eV), C–N (286.28 eV), C–O/C=O (288.99 eV), and π–π* transition (292.42 eV) transitions can be distinguished in the C1s spectra of NC–800 and Fe–NC–800, respectively (Figure 3B,E) [24]. Because C–N was next to Fe–Nx and the C–N bond preferentially fractured, the quantity of C–N species in Fe–NC–800 reduced when compared to NC–800, which improved the catalytic performance [25]. The fact that the iron–doped material forms Fe–Nx active sites can be inferred from the N1s spectrum in Figure 3F, which can be divided into pyridinic–N (398.1 eV), pyrrolic–N (399.5 eV), graphitic–N (401.6 eV), and oxynitride (403.9 eV) [26]. The characteristic peak of pyrrole N of Fe–NC–800 is shifted in position compared to NC–800, indicating that the addition of Fe has changed the coordination environment of nitrogen, which may be caused by the coordination of pyrrole N with Fe to form Fe–Nx sites [27]. The two main configurations of N1s of Fe–NC–800 are pyridine nitrogen and graphite nitrogen. By increasing the electron density and other doping factors, pyridine nitrogen can enhance the catalytic activity of electrode materials, while graphite nitrogen is thought to improve the electron transfer ability of carbon–based materials [28,29]. Meanwhile, pyrrole nitrogen is believed to enhance the catalytic activity by reducing the carbon band gap energy, as it usually shows fast charge mobility [30]. Nitrogen content falls when pyrolysis temperature rises (Table S1), which is caused by the material’s structure being destroyed and nitrogen atoms escaping as N_2_, HCN, NH_3_, or other gases [31]. The Fe2p XPS spectra of Fe–NC–800 is shown in Figure 3D. The peaks at 710.91 eV, 722.78 eV, 717.56 eV, and 732.47 eV correspond to Fe^2+^, and the peaks at 714.65 eV, 726.69 eV, 720.94 eV, and 734.83 eV correspond to Fe^3+^ [21]. The high resolution Fe2p spectrum of Fe–NC–800 shows a weak signal of Fe^0^ (Fe_3_C) at 707.72 eV, which may be due to the encapsulation of nanoparticles by the graphite carbon layer [32].

### 2.2. Electrochemical Behavior of Fe–NC/GCE

The cyclic voltammetry (CV) curve in the 5.0 mM K_3_[Fe(CN)_6_ standard solution containing 0.1 M KCl is shown in Figure S2A. As can be seen, Fe–NC–800 has a much higher current response than bare GCE, NC–800/GCE, Fe–NC–700/GCE, and Fe–NC–900/GCE. Additionally, the peak potential difference is the smallest, indicating that Fe–NC–800 has the fastest electron transfer [33]. Combined with the XRD, TEM, and Raman test results mentioned previously, it can be inferred that the doping of Fe and the formation of Fe/Fe_3_C nanoparticles can promote the charge transfer ability of the nitrogen–doped carbon material, which contributes to the electrocatalytic performance of the modified electrode.

**Figure 4:**
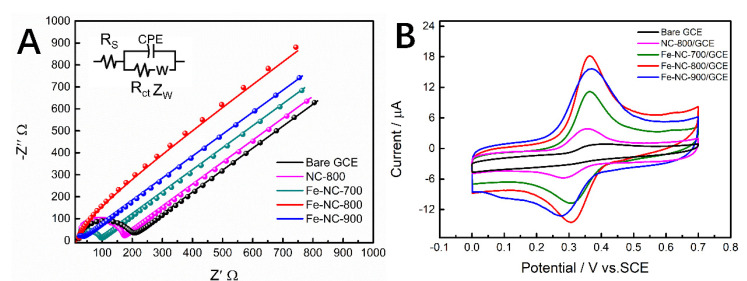
(**A**) Electrochemical impedance maps (EIS) of electrodes modified with different materials; (**B**) CV plots of electrodes modified with different materials in 0.1M CPB solution (pH = 6.8) containing 50 μM acetaminophen, scan rate 100 mV/s.

The electrochemical impedance (EIS) fitted circuit uses a Randles–Warburg circuit [Rs(CPE[RctZ_W_])] fitted with a constant phase element (Figure 4A), where Rs is the solution resistance, Rct is the charge transfer resistance, CPE is the constant phase element, and Z_W_ is the Warburg impedance. Here, the CPE is used instead of the capacitance Q to describe the non–ideal behavior of the system caused by the rough or porous surface of the material, and the specific expression for the CPE is Z_CPE_ = ((jω)^a^Y_0_)^−1^, a constant phase element index, in the range from 0~1, Y_0_ = capacitance. The value of CPE–P for Fe–NC–800/GCE was found to be 0.87 after fitting, which is between 0.5 and 1. This indicates that there is a dispersion effect on the electrode surface. The capacitive resistance arc of the bilayer appears in the high frequency part, while the Warburg impedance appears in the low frequency part, indicating that the diffusion control exceeds the electrochemical control. The fitted data yielded charge transfer resistances (Rct) of 187.4 Ω, 164.9 Ω, 105.4 Ω, 10.6 Ω, and 34.7 Ω for Bare GCE, NC–800/GCE, Fe–NC–700/GCE, Fe–NC–800/GCE, and Fe–NC–900/GCE, respectively. The charge transfer resistance is inversely proportional to the capacitance [34]. The value of Rct confirms the conductive nature of Fe–NC–800, which promotes a faster electron transfer [35], in agreement with the CV data in Figure S2A.

Figure S2B shows CVs of various electrodes in 0.1 M KCl containing 5.0 mM K_3_[Fe (CN)_6_]. It is clear that the oxidation peak charge of Fe–NC–800/GCE is linearly related to the square root of the scan rate, according to the Randles–Sevcik equation [36]:

*I_p_* = 2.69 × 10^5^
*n*^3/2^*AD*^1/2^*Cv*^1/2^ (1)

where *A* is the effective surface area, n is the number of electrons transferred (*n* = 2), *D* is the diffusion coefficient of K_3_[Fe(CN_6_)] (7.6 × 10^−6^ cm^2^/s), and *C* is the bulk concentration of K_3_[Fe(CN_6_)]. The effective surface area of Fe–NC–800/GCE can be calculated to be 0.285 cm^2^, which is much higher than that of bare GCE (0.023 cm^2^), NC–800/GCE (0.068 cm^2^), Fe–NC–700/GCE (0.143 cm^2^), and Fe–NC–900/GCE (0.201 cm^2^). Combined with Raman results, it can be seen that Fe–NC–800 has the highest defect degree, thus, exposing more active sites, so it has the largest effective surface area.

The electrochemical behavior of AC at bare GCE, NC–800/GCE, Fe–NC–700/GCE, Fe–NC–800/GCE, and Fe–NC–900/GCE were investigated by CV (Figure 4B). In 0.1 M CPB (pH = 6.8) containing 50 µM AC (scan rate 100 mV/s), only a weak oxidation peak (0.416 V) was observed on bare GCE, indicating that the redox of AC on bare GCE is irreversible. NC–800/GCE showed only a pair of weak redox peaks, indicating that the MF resin–derived nitrogen–doped carbon is not sufficient for the catalytic ability of AC. A pair of obvious oxidation peaks (0.364 V) and reduction peaks (0.308 V) appeared on Fe–NC–800/GCE. This indicates that the higher effective active area and increased reactive sites of the material due to Fe–doping can promote the catalytic ability of nitrogen–doped carbon for AC. We speculated that AC was oxidized to N–acetyl–4–benzoquinone imine at the oxidation peak. The redox peak current of Fe–NC–800 is approximately four times that of NC–800, and the peak potential difference (58 mV) of NC–700 is greater than that of Fe–NC–800 (56 mV), indicating that Fe–doping can improve the electron transfer rate of materials and provide more active sites. The peak potential difference between Fe–NC–800 and Fe–NC–700 was almost the same, but when the pyrolysis temperature reached 900 °C, the peak potential difference increased to 63 mV. This was because the temperature is too high, resulting in the destruction of the material structure and the decrease in the defect degree, resulting in the reduction of the exposed active sites. In general, the reason why Fe–NC–800 has the best catalytic activity can be explained as follows: (1) Fe–doping forms Fe–Nx active sites and Fe/Fe_3_C nanoparticles in the material, which improves the electron transfer efficiency of the material; (2) appropriate pyrolysis temperature makes the material have a high defect degree and exposes more active sites.

**Figure 5:**
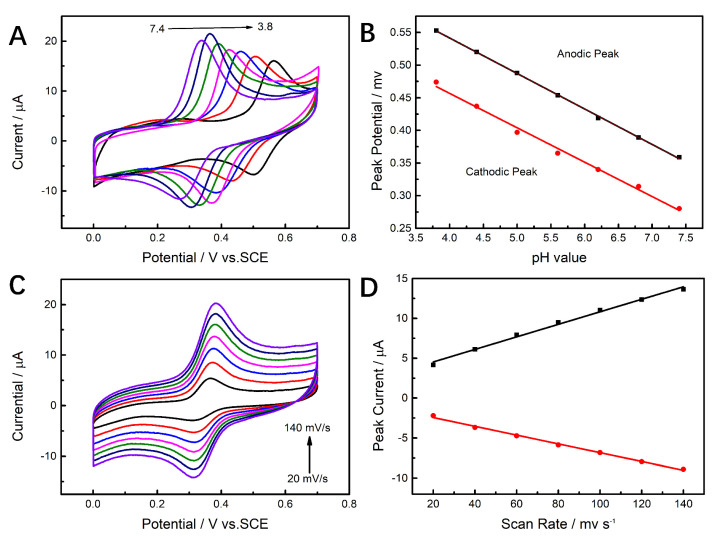
CVs of Fe–NC–800/GCE in 0.1 mol/L CPB with 50 µM AC (scan rate 100 mV/s) of different pH (**A**); effect of pH value on the oxidation peak potential (**B**); CV of Fe–NC–800/GCE in 0.1 mol/L CPB (pH = 6.8) with 50µM AC at different scan rates (20–140 mV/s) (**C**); the linear dependence plot of scan rate on peak currents (**D**), the black line represents the oxidation peak current and the red line represents the reduction peak current.

When it comes to the working electrode and the sensing of the target, the supportive electrolyte’s pH has a considerable impact. Therefore, CV was used to analyze the effects of pH on the peak current and potential of oxidation of AC (50 μM), with 0.1 M CPB and a constant scan rate of 100 mV/s, the pH value was in the range from 3.8–7.4. As can be seen from Figure 5A, as the pH value rises from 3.8 to 7.4, the redox peak potential of AC shifts in the opposite direction, indicating that protons are involved in the redox reaction of AC [37]. This effect might be caused by the –NHCOCH_3_ and –OH groups of AC molecules changing structurally in various pH solutions [38]. Additionally, pH = 6.8 was found to produce the strongest electrochemical signal, thus, it was decided that this was the best pH for detecting AC in following studies. Figure 5B illustrates the good linear relationship between the oxidation and reduction peak potentials and pH. The linear regression and correlation coefficient equations derived from the linear plots of redox peak potential and pH are obtained as follows:

*E_pa_* = −0.05435 pH + 0.7589 (*E_pa_: V*, R^2^ = 0.9993) (2)

*E_pc_* = −0.05268 pH + 0.6674 (*E_pc_: V*, R^2^ = 0.9922) (3)

**Scheme 1:**
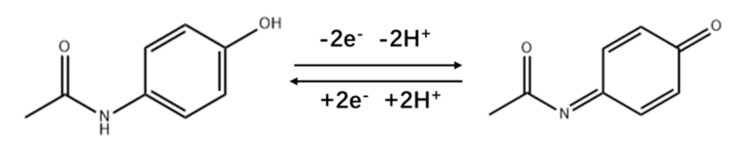
Mechanisms of AC Electrochemical Reactions.

It is possible to determine the link between *E_pa_* and *E_pc_* and pH value; the slopes are 54.4 mV pH^−1^ and 52.7 mV pH^−1^, respectively. These values are both quite close to 59 mV pH^−1^. This suggests that both protons and electrons are present in equal amounts during the reaction [11]. The proton number (*m*) was calculated to be 2 using Equation (4) [39]:

d*E*_p_/d*pH* = 2.303 *m*RT/2F (4)

where T is the temperature, m is the number of protons, R is the ideal gas constant, and F is the Faraday constant. Therefore, it may be concluded that the redox reaction between AC and Fe–NC/GCE involves two electrons and two protons. In other words, AC is oxidized to N–acetyl–4–benzoquinoneimine at the oxidation peak, and N–acetyl–4–benzoquinone imine is reduced to AC at the reduction peak, and so on repeatedly to form a catalytic cycle, as shown in Scheme 1.

We used CV to examine the impact of scan rate on oxidation current in order to better understand the kinetic of AC oxidation on Fe–NC–800 electrodes. Scan rates varied between 20 and 140 mV/s when 50 μM AC was present in CPB (pH = 6.8). The oxidation peak current (*I_pa_*) and the reduction peak current (*I_pc_*), which are both depicted in Figure 5C, both gradually rise with an increase in scan rate. The correlation between *I_pa_*, *I_pc_*, and scan rate is depicted in Figure 5D. They all have a good linear relationship, as can be seen:

*I_pa_* = 0.07868 *v* + 2.947 (R^2^ = 0.9969) (5)

*I_pc_* = −0.05487 *v* − 1.3384 (R^2^= 0.9981) (6)

The linear correlation between current and scan rate suggests, in accordance with Equations (5) and (6), that the redox reaction of AC on Fe–NC–800/GCE is a surface–controlled process as opposed to a diffusion–controlled activity [40].

Figure S3A shows the change trend in AC oxidation peak current with different volumes of ink composed of 2 mg Fe–NC–800, 1 mL water, and 5 μL 5 wt% Nafion solution. The oxidation peak current increases with the increase in the volume of dripping ink from 1 μL to 5 μL. This result may be due to the gradual increase in Fe–NC–800 content on the surface of the modified electrode as the volume of the dropwise added material increases from 1 μL to 5 μL, which will provide more active catalytic sites and, therefore, the catalytic performance of the electrode is gradually enhanced, and the response current is gradually increased. The oxidation peak current decreases gradually when the volume exceeds 5 μL. Speculatively, the phenomenon observed could be attributed to the abundant accumulation of Fe–NC–800 on the electrode surface. This build–up may excessively raise the contact resistance, thereby reducing the electrode’s current response. The reason for this phenomenon, we speculate that it is due to the accumulation of too much Fe–NC–800 on the electrode surface, which instead leads to some catalytically active sites being covered, making the modified electrode less catalytically active. Therefore, the optimized ink volume is 5 μL. Because the kinetic process of the electrochemical reaction of AC on Fe–NC–800/GCE is controlled by adsorption, the effect of the enrichment time needs to be considered. The effect of Fe–NC–800/GCE on the peak oxidation current of 50 µM AC (scan rate 100 mV/s) at different enrichment times was investigated by DPV (Figure S3B). The oxidation peak current gradually increased by increasing the enrichment time from 0 s to 180 s, reaching the maximum value at 180 s. Further extension of the enrichment time resulted in essentially no change in the oxidation peak current, which indicates that AC basically reached saturation at the Fe–NC–800/GCE surface at 180 s. Therefore, an enrichment time of 180 s was used in the subsequent experiments.

**Figure 6:**
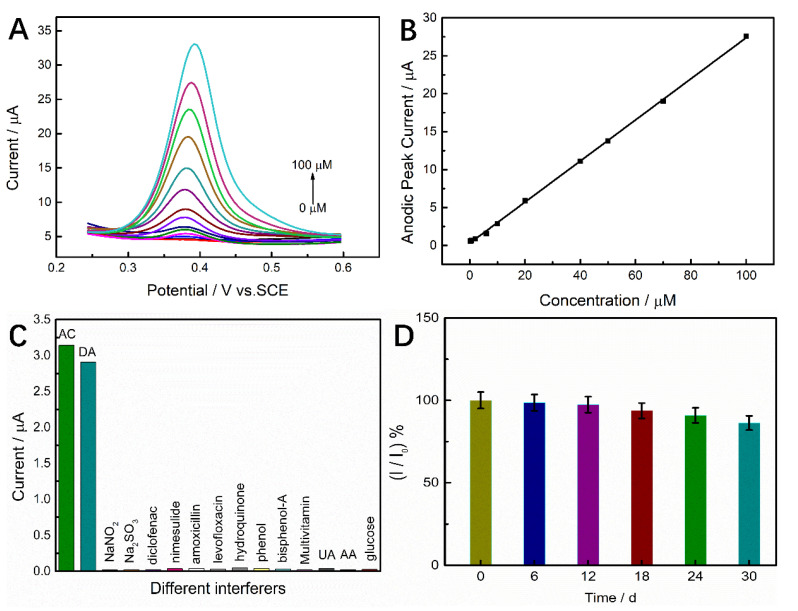
(**A**) DPV curves of Fe–NC–800/GCE with different concentrations of AC (0–100 μM) in 0.1 mol/L CPB (pH = 6.8), different color lines in the graph represent the DPV curves with different concentrations of AC added. (**B**) The corresponding calibration curve of AC concentration and current. (**C**) Response currents of Fe–NC–800/GCE in 0.1 mol/L CPB (pH = 6.8) to 10µM AC and ten times the concentration of interferents (uric acid (UA), glucose, dopamine (DA), ascorbic acid (AA), sodium nitrite, sodium sulfite, diclofenac, nimesulide, amoxicillin, levofloxacin, hydroquinone, bisphenol A, phenol, multivitamin tablets), different color columns represent the response current of Fe–NC–800/GCE to AC and different interferents (**D**) Long–term stability experiment of AC sensor based on Fe–NC–800 (0–30 d), the different color columns represent the response current of Fe–NC–800/GCE to the same con-centration of AC after the current number of days of placement.

Differential pulse voltammetry (DPV) has high sensitivity, making it possible to detect even low concentrations of analytes [40]. DPV is capable of providing better resolution compared to other voltammetric techniques, as it minimizes the impact of the background noise on the signal [41]. We, therefore, utilized DPV to quantitatively detect AC in 0.1 M CPB. The pulse width of DPV is 0.2 s, the pulse period is 0.5 s, the potential increment is 4 mV, and the voltage range is from 0.2–0.6 V [42]. As shown in Figure 6A, when AC concentration rises, a distinct increase in peak DPV current can be seen. As depicted in Figure 6B, a good linear correlation between peak current and concentration was obtained in the range from 0–100 μM. The linear regression equation was *I* (μA) = 0.27076 *c* (μM) + 0.30269 (R^2^ = 0.9997). The LOD was calculated to be 0.026 μM via 3σ/S, where 3 represents the confidence factor and σ is the standard deviation of the blank sample, we measured 20 times to obtain the average value as σ, and S is the slope of the fitted straight line [43]. We used the same method to perform DPV tests on bare GCE, NC–800/GCE, Fe–NC–700/GCE, and Fe–NC–900/GCE, and the results are shown in Figure S5 and Table S2. It can be seen that the sensitivity and detection limits of Fe–NC–800/GCE are better than those of bare GCE, NC–800/GCE, Fe–NC–700/GCE, and Fe–NC–900/GCE.

It can be seen that the LOD of Fe–NC–800/GCE for AC is lower than many non–noble metal catalysts in recent years, which is close to the report of some noble metal catalysts (Table 1). The factors listed below may be responsible for the good electrochemical performance: (1) the aromatic conjugated electron cloud of carbon and the aromatic electron cloud of acetaminophen interact to give the outer carbon a strong adsorption capacity for AC [44]; (2) the formation of Fe/Fe_3_C nanoparticles can promote the catalytic activity of Fe–Nx, improve the material’s efficiency at transferring electrons, and shorten the electron transfer path of the redox reaction of AC on Fe–NC–800/GCE; (3) Fe–doping enhances the carbon material matrix’s degree of defect and exposes additional active sites. The outcomes demonstrate the reliability of the Fe–NC–800/GCE, presented in this paper, for detecting AC current.

### 2.3. Repeatability, Stability, and Selectivity

Selectivity is an important parameter for evaluating the sensor performance. To assess the selectivity of Fe–NC–800/GCE, we tested anti–interference substances with DPV (Figure 6C), with the methods refering to the relevant literature [53]. We used uric acid (UA), glucose, dopamine (DA), ascorbic acid (AA), sodium nitrite, sodium sulfite, diclofenac, nimesulide, amoxicillin, levofloxacin, hydroquinone, bisphenol–A, phenol, and multivitamin tablets as interfering substances. The above interfering substances were tested with DPV. After testing, Fe–NC–800/GCE was found to have no significant current response to UA, AA, glucose, sodium nitrite, sodium sulfite, diclofenac, nimesulide, amoxicillin, levofloxacin, hydroquinone, bisphenol A, phenol, and multivitamins. Although it reacted to DA, the oxidation peak potentials of AC and DA were far apart as seen by the DPV curve (Figure S6), and the presence of DA had almost no effect on the reaction current of AC, which indicates that the sensor has a good selectivity for the detection of AC.

We adopted DPV to detect the reproducibility of Fe–NC–800/GCE, fabricated five different samples of Fe–NC–800/GCE to detect AC in 0.1 M CPB (pH = 6.8) and observed that the current responses of the modified electrodes were almost the same for five times (Figure S8A,B). We continuously measured the same Fe–NC–800/GCE five times (Figure S8C) and found that the response current did not change significantly, indicating that Fe–NC–800/GCE had good repeatability. The single electrode was stored for 30 days, and the same concentration of AC was detected every 6 days. After 30 days, the electrochemical sensor showed acceptable stability, with a peak current retention of approximately 86.3% (Figure 6D). This structure of nanoparticles wrapped by a graphitic carbon layer avoids the direct contact between the active center and the reaction medium, which greatly reduces the carbon corrosion and iron loss in the Fe–NC–800 catalyst and improves the stability of the material.

### 2.4. Real Sample Studies

We tested the AC concentration in commercially available drugs using a standard addition method to verify the practical application of the sensor, the methodology with reference to the relevant literature [33]. Commercially available tablets were ground in a mortar and pestle, dissolved in 10 mL of ethanol, centrifuged, and the supernatant diluted to 250 mL with 0.1 M CPB (pH = 6.8) solution. Then, 1 mL of the solution (acetaminophen tablet solution) and 9 mL of 0.1 M CPB (pH = 6.8) solution were used to form the solution to be tested. The AC in the solution to be measured was measured to be 12.7 µM. A standard solution of known concentration of AC was then added to it and the feasibility of the proposed sensor was assessed by calculating the recovery. The results are shown in Table 2. The recoveries of all spiked samples ranged from 96.1% to 102.2%, indicating that Fe–NC–800/GCE is an efficient and reliable sensing platform for the detection of AC in real samples.

## 3. Experimental

### 3.1. Reagents

Acetaminophen was from Sinopharm Shanghai, China. Melamine (99%), formaldehyde (37%), citric acid (99%), disodium hydrogen phosphate (99%), ferric chloride hexahydrate (99%), polyvinyl alcohol (98 %, Mw~47,000), uric acid (UA) (99%), glucose (99%), dopamine (DA) (98%), ascorbic acid (AA) (99%), diclofenac (98%), hydroquinone (99%), and bisphenol A (99%) were from Macklin Biochemical Technology Co., Ltd. (Shanghai, China). All reagents were used without further purification. Citrate phosphate buffer (CPB), which served as a supporting electrolyte, was made from a solution of citric acid (0.1 M) and sodium phosphate (0.2 M).

Acetaminophen tablets (500 mg/tablet) were from Sino–American Tianjin SmithKline and French Lab., Ltd. (Tianjin, China). Nimesulide (100 mg/tablet) was from Beijing Yongzheng Pharmaceutical Co., Ltd. (Beijing, China). Amoxicillin (250 mg/tablet) was from Cspc Heibei Zhongrun Pharmaceutiacl Co., Ltd. (Hebei, China). Levofloxacin (100 mg/tablet) was from Shandong Lukang Group Saite Co., Ltd. (Xintai, China). Multivitamin tablets (Vitamin B6 0.25 mg/tablet and Vitamin B1 2.5 mg/tablet) were from Hangzhou Minsheng Healthcare Co., Ltd. (Zhejiang, China).

### 3.2. Synthesis of Fe–NC

**Scheme 2:**
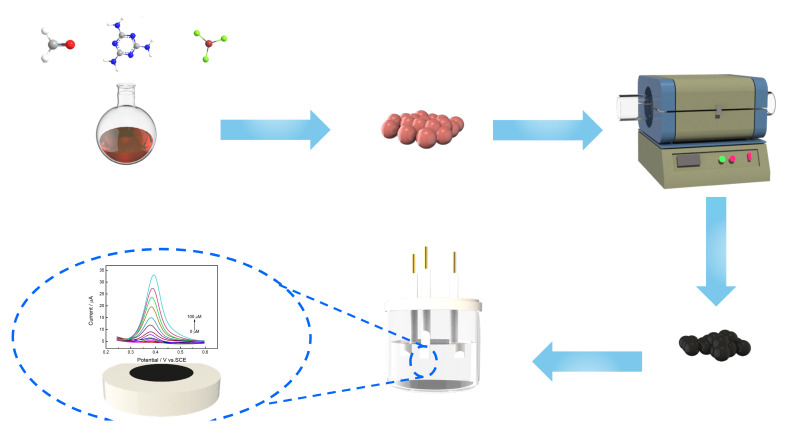
The preparation process of Fe–NC and its schematic diagram for the detection of acetaminophen.

The experimental procedure is shown in Scheme 2, to create Solution A, 90 mL of water was used to dissolve 0.6 g of polyvinyl alcohol (PVA) (solid, 98%, Mw~47,000). Solution B was created by thoroughly stirring 3.78 g of melamine into 30 mL of water, then adding 10 mL of formaldehyde (37%). Solution B was heated in a 65 °C water bath until it was clear and achromic. After adding 1 g of FeCl_3_·6H_2_O (solid, 99%), solution B was heated in a 65 °C water bath for another 30 min. Next, solution A was added. The mixture was then reacted for another 30 min, then centrifuged and dried to produce the polymer powder. The samples were then pyrolyzed for 2 h at 800 °C with a heating rate of 5 °C min^−1^ in a nitrogen atmosphere, and then washed with 500 mL of 0.5 mol/L 0.5 M H_2_SO_4_ to remove any remaining residue. The finished product was then labeled as iron/nitrogen–doped carbon after being neutralized with deionized water (Fe–NC–800). The same procedure was used to create samples with pyrolysis temperatures of 700 °C and 900 °C, which were referred to as Fe–NC–700 and Fe–NC–900, respectively. Additionally, using the same manufacturing method, nitrogen–doped carbon without Fe was also produced, this material is known as NC–800.

### 3.3. Characterizations

A Quanta250 (Thermo Fisher Scientific Co., Ltd., USA) field emission environmental scanning electron microscope was used to capture images of the samples under a scanning electron microscope (SEM). Using an FEI Tecnai G2T20 (Thermo Fisher Scientific Co., Ltd., USA), 200 kV measurements were made on the sample’s transmission electron microscopy (TEM) pictures. The sample’s Raman spectrum was captured by the WJGS–034 Micro Raman Spectrometer at a wavelength of 532 nm(Horiba(China)Trading Co., Ltd., France). Thermo Scientific K–Alpha+ was used to conduct X–ray Photoelectron Spectroscopy (XPS) (Thermo Fisher Scientific Co., Ltd., USA). The X–ray diffraction patterns (XRD) of the samples were obtained by a Rigaku Ultima Ⅳ X–ray diffractometer (Rigaku Co., Ltd., Japan). The deposition thickness of samples on glassy carbon electrodes were determined using a 37XC–PC inverted microscope.

A CHI660E electrochemical workstation (Shanghai Chenhua Instrument Co., Ltd., Shanghai, China) was used in this work coupled with a Three–Electrode System. Pt and saturated calomel electrodes served as the counter and reference electrodes (SCE), respectively. GCE (*d* = 3 mm) or Fe–NC composite–modified GCE was used as the working electrode. Prior to the modification process, the GCE was sequentially polished with 1 mm, 0.3 mm, and 0.05 m alumina/water slurry. Additionally, homogeneous ink containing 2 mg Fe–NC, 1 mL water, and 5 μLNafion (5 wt%) was obtained via a subsequent ultra–sonication treatment of 30 min. An Fe–NC modified electrode was prepared via drop casting method with 5μL of ink. The electrode was stored under N_2_ atmosphere at room temperature (~25 °C). All electrochemical tests were carried out at a constant temperature (25 °C). The thickness of the Fe–NC–800 layer deposited on GCE was measured to be 38.2 µm.

## 4. Conclusions

In this report, Fe–NC nanomaterials were prepared by one–step in situ pyrolysis using MF as raw material. The relationship between pyrolysis temperature, microstructure, and electrochemical performance was analyzed, and it was successfully used for electrochemical detection of AC. During the pyrolysis process, a large number of triazine groups in MF provide abundant nitrogen as the anchor site for Fe, forming the Fe–Nx active center. Part of Fe–Nx was reduced to Fe_3_C during the pyrolysis process. Fe–NC–800 has the best analytical performance for AC due to the high catalytic activity of Fe–Nx sites formed in the material, the promotion of Fe/Fe_3_C on Fe–Nx, the highest defect degree, and the excellent stability and selectivity of Fe/Fe_3_C structure wrapped by graphite carbon. With these excellent characteristics, the designed electrode can be recommended for future analytical applications to detect AC. Despite some limitations, our research offers insights into the use of the Fe_3_C/Fe–Nx composite as an electrode material for electrochemical sensing and catalysis. To further enhance our understanding, we plan to investigate the impact of the coordination state of Fe species on electrochemical properties, and to develop an effective masking method to differentiate Fe_3_C from Fe–Nx and elucidate their cooperative action. Moreover, we aim to extend our application scope beyond acetaminophen by exploring the simultaneous detection of multiple analytes, such as dopamine and acetaminophen, to expand the versatility and utility of our sensing platform. These efforts will augment our fundamental knowledge and practical application of the Fe_3_C/Fe–Nx composite in electrochemical sensing and catalysis.

## Figures and Tables

**Table 1:** Comparison of LOD of various AC electrochemical sensor.

Materials (Method)	Linear Range	LoD	Analytical Methods	Ref.
P–NC ^a^/GCE	3–110 μM	0.5 μM	DPV	[1]
PPy/PPa–PGE ^b^	0.2–500 μM	0.89 μM	DPV	[45]
N,S–doped C@Pd nanorods	0.033–120 μM	0.011 μM	DPV	[46]
ILC–CPE ^c^	0–120 μM	2.8 μM	DPV	[47]
IL–NH_2_–Fe_3_O_4_NP–MWCNT ^d^–GCE	0–0.7 μM	0.4 μM	DPV	[48]
MoS_2_–TiO_2_/rGO/SPE	0.1–125 μM	0.046 μM	DPV	[49]
Pd–SB/GCE	0–50 μM	0.067 μM	CV	[50]
TFPB–BD–COF/caCTF–1–700/COOH–MWCNT/GCE ^e^	0.6–150 μM	0.053 μM	DPV	[51]
Au–PEDOT ^f^/rGO/GCE	0.001–8000 μM	0.001 μM	i–t ^g^	[52]
Fe–NC–800/GCE	0–100 μM	0.026 μM	DPV	This work

^a^, P–NC: nitrogen–rich porous carbon. ^b^, PPy/PPa: polypyrrole/polypyrrole–3–carboxylic acid copolymer; PGE: pencil graphite electrode. ^c^, ILC: ionic liquid crystal; CPE: carbon paste electrode. ^d^, IL: 1–ethyl–3–methylimidazolium tetrafluoroborate ionic liquid; NH_2_–Fe_3_O_4_NP: amino group functionalized magnetite nanoparticles; MWCNT: multi–walled carbon nanotubes. ^e^, TFPB–BD–COF/caCTF–1–700/COOH–MWCNT: Covalent Organic Frameworks and Covalent Triazine Frameworks Binding via Carboxylated Multiwalled Carbon Nanotubes. ^f^, PEDOT: poly(3,4–ethylenedioxythiophene). ^g^, i–t: chronoamperometry.

**Table 2:** Determination of paracetamol in pharmaceutical samples.

Sample	Detected (µM)	Spiked (μM)	Found (μM)	Recovery (%)
Paracetamol Tablet (500 mg/tablet)	12.7	10	23.2	102.2
		30	41.8	97.9
		50	60.3	96.1

## Data Availability

Data are contained within the article or Supplementary Materials. The data sets generated during and/or analyzed during the current study are available from the corresponding author upon rea-sonable request.

## Supplementary Materials

The following supporting information can be downloaded at: https://www.mdpi.com/article/10.3390/molecules28073006/s1, Reagent safety and toxicity analysis. Figure S1. SEM image of MF resin (A) and NC–800 (B). Figure S2. (A) CV curves of 5.0 mM [Fe(CN)6] solution in the presence of 0.1 M KCl; (B) Fe–NC–800/GCE, (C) bare GCE, (D) NC–800/GCE, (E) Fe–NC–700/GCE, (F) Fe–NC–900/GCE in the presence of 0.1 M KCl 5.0 mM [Fe(CN)6] CV curves at different scan rates in solution. Figure S3. Effect of dropwise material volume (A) and enrichment time (B) on AC oxidation peak current (Folding Line Chart). Figure S4. Effect of dropwise material volume (A) and enrichment time (B) on AC oxidation peak current. Figure S5. Bare GCE (A,B), NC–800/GCE (C,D), Fe–NC–700/GCE (E,F), Fe–NC–900/GCE (G,H) under the same conditions for DPV testing. Figure S6. Interference experiment of the Fe–NC–800 measured with AC and interferents(uric acid(UA), glucose, dopamine(DA), ascorbic acid(AA), sodium nitrite, sodium sulfite, diclofenac, nimesulide, amoxicillin, levofloxacin, hydroquinone, bisphenol–A, phenol, mul-tivitamin) in 0.1 mol/L CPB (pH = 6.8). Figure S7. Long–term stability experiment of AC sensor based on Fe–NC–800/GCE (0–30 days). Figure. S8 Different Fe–NC–800/GCE to detect the current response of AC (50μM) in 0.1 M CPB (pH = 6.8), (A) DPV; (B) Response current histogram; (C) Five measurements of the same electrode in 0.1M CPB (pH = 6.8). Figure S9. The DPV curve of paracetamol in the drug was determined in 0.1 MCPB (pH = 6.8). Figure S10 The thickness of the Fe–NC–800 layer deposited on GCE. Table S1. C, N and Fe contents of Fe–NC group calculated from XPS results. Table S2. Com-parison of sensitivity and detection limits of electrodes modified with different materials.
