# Supplementary material for: Fe–Decorated Nitrogen–Doped Carbon Nanospheres as an Electrochemical Sensing Platform for the Detection of Acetaminophen
Source: Molecules. 2023 Mar 28;28(7):3006. doi: 10.3390/molecules28073006 (PMC10095704; doi:10.3390/molecules28073006)
Supplement: Supplementary file 1 [file molecules-28-03006-s001.zip › molecules-2237172-supplementary.pdf]

Fe-decorated nitrogen-doped carbon nanospheres as a electrochemical sensing platform for the detection of acetaminophen

Xiangchuan Zhao [1, 2], Liping Zhang[1], Zhaoyun Chu[1], Qing Wang[1], Yue Cao[1], Jun Cao[1], Jiao Li[1], Wu Lei [3], Boming Zhang [2], Weimeng Si [1,]*

[1] School of Materials Science and Engineering, Shandong University of Technology, Xincunxi Road 266th, Zibo, 255000, China

[2] Institute of Advanced Materials, Shandong Institutes of Industrial Technology, Jinan, 250000, China

[3] School of Chemistry and Chemical Engineering, Nanjing University of Science and Technology, Xiaolingwei 200th, Nanjing, 210094, China

Reagent safety and toxicity analysis

Polyvinyl alcohol: This product is non-hazardous, no explosion, no oxidation, no corrosion, no radioactivity, non-toxic and harmful.

Melamine: White crystals. Heating sublimation. Not combustible. It is soluble in methanol, formaldehyde, acetic acid, ethylene glycol, glycerol, pyridine, insoluble in ether, benzene, carbon tetrachloride. It can decompose to produce highly toxic cyanide gas at high temperature.

Formaldehyde: This product is colorless or almost colorless clear liquid, irritating odor, can stimulate the nose and throat mucosa ; turbidity is easy to occur in the cold place.

Ferric chloride hexahydrate: Yellow-brown crystal or block solid, odorless, astringent. The melting point is 37 °C. The boiling point is 280 ~ 285 °C. It is soluble in water, ethanol and ether. Its aqueous solution is strongly acidic, which can solidify the protein. It is deliquescent and can be deliquescent into reddish brown liquid in the air. Non-combustible, corrosive, can stimulate the skin, eyes. Inhalation can cause sore throat, abdominal pain, diarrhea, nausea, etc.

Some reagents required for the preparation of the sensor are dangerous when the experimental operation is improper, but the correct experimental operation will not cause danger. After the material is carbonized, the toxic substances will volatilize or be converted into non-toxic and safe carbon materials.

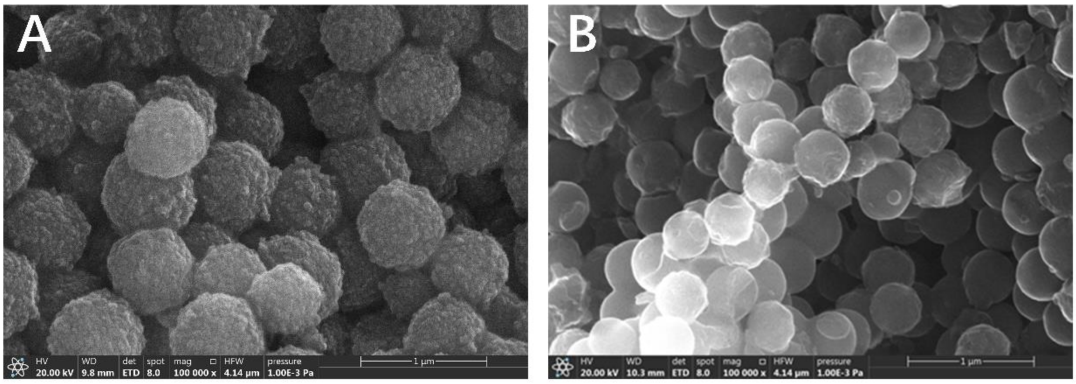

Fig S1. SEM image of MF resin(A) and NC-800(B).

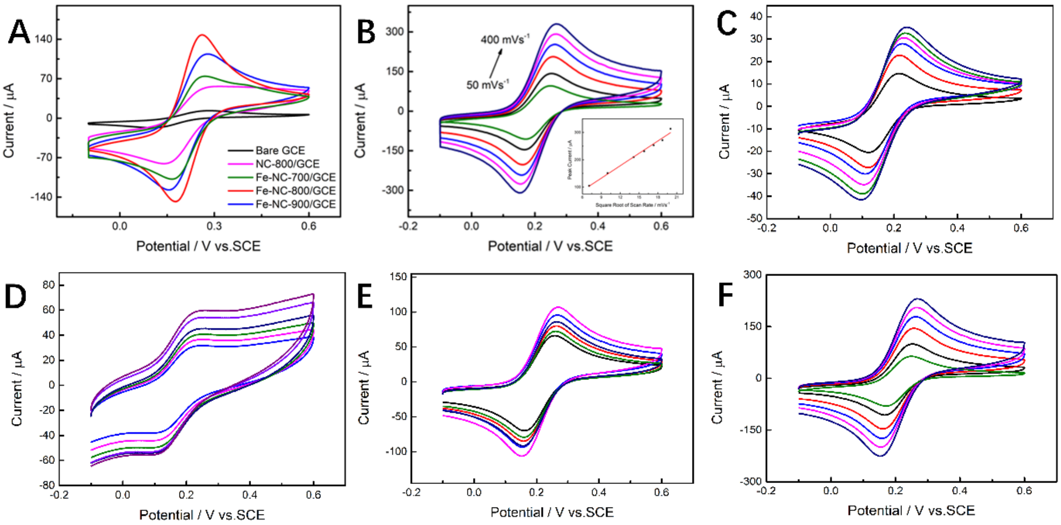

Fig S2. (A) CV curves of 5.0 mM [Fe(CN)$_6$] solution in the presence of 0.1 M KCl ; (B) Fe-NC-800/GCE,(C)bare GCE, (D)NC-800/GCE, (E)Fe-NC-700/GCE,(F)Fe-NC-900/GCE in the presence of 0.1 M KCl 5.0 mM [Fe(CN)$_6$] CV curves at different scan rates in solution.

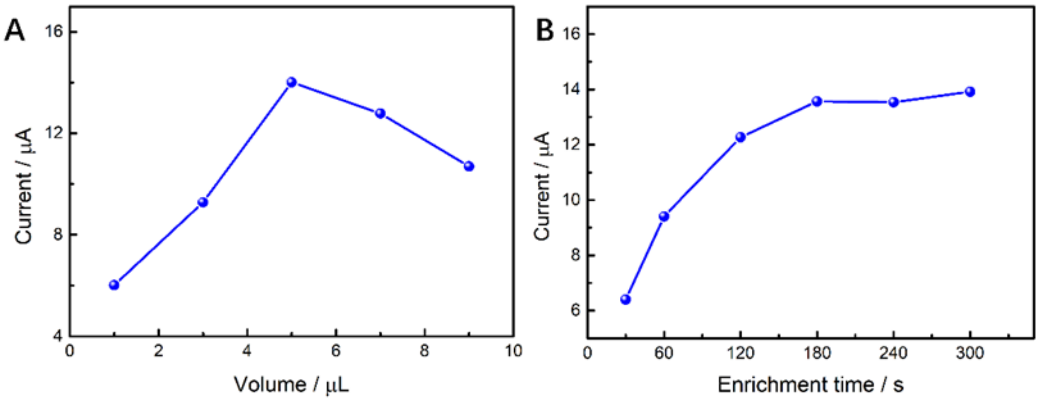

Fig S3. Effect of dropwise material volume (A) and enrichment time (B) on AC oxidation peak current (Folding Line Chart).

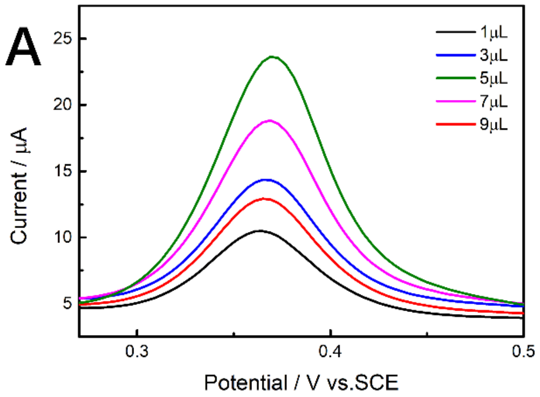

Fig S4. Effect of dropwise material volume (A) and enrichment time (B) on AC oxidation peak current.

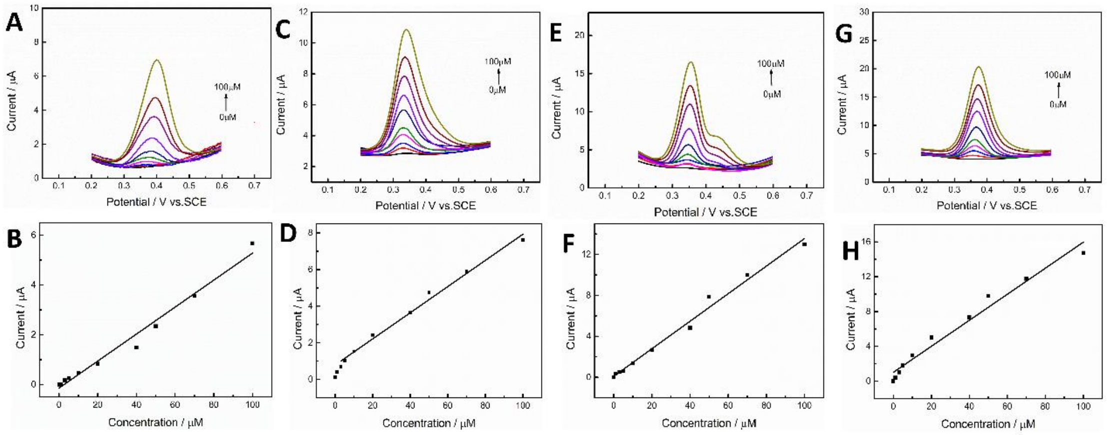

Fig S5. Bare GCE (A, B), NC-800/GCE (C, D), Fe-NC-700/GCE (E, F), Fe-NC-900/GCE (G, H) under the same conditions for DPV testing.

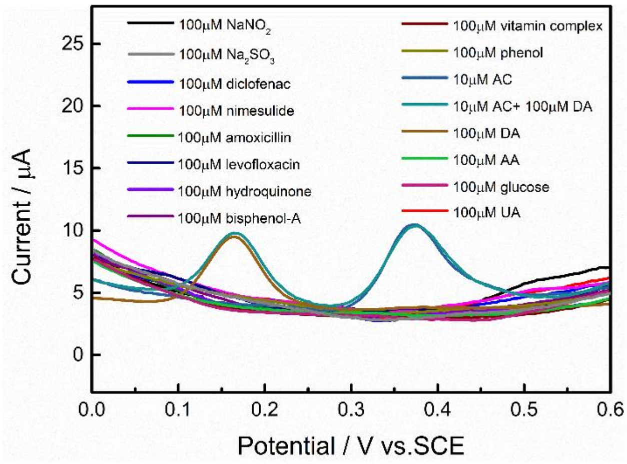

Fig S6. Interference experiment of the Fe-NC-800 measured with AC and interferents(uric acid(UA), glucose, dopamine(DA), ascorbic acid(AA), sodium nitrite, sodium sulfite, diclofenac, nimesulide, amoxicillin, levofloxacin, hydroquinone, bisphenol-A, phenol, multivitamin) in 0.1 mol/L CPB(pH=6.8)

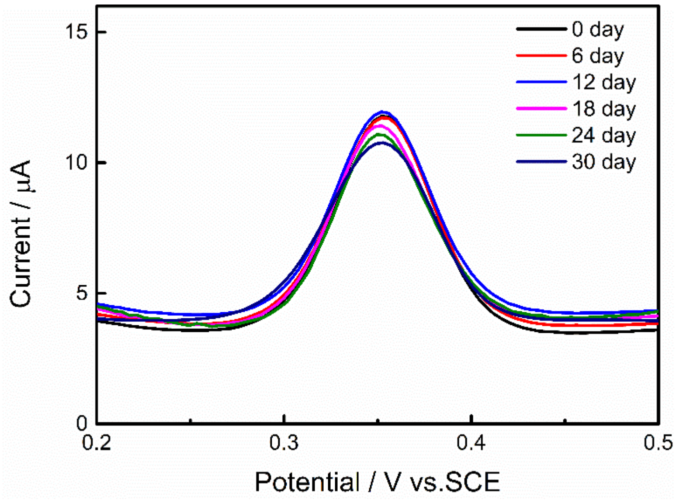

Fig S7. Long-term stability experiment of AC sensor based on Fe-NC-800/GCE (0-30 days).

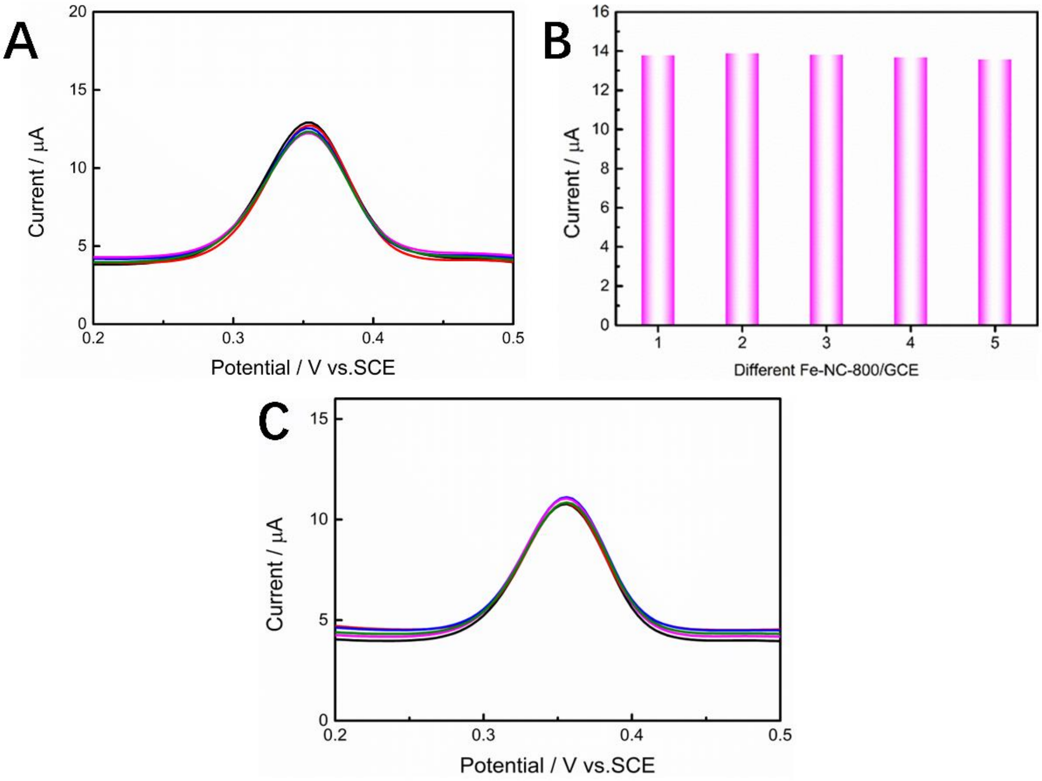

Fig. S8 Different Fe-NC-800/GCE to detect the current response of AC(50μM) in 0.1M CPB (pH=6.8),(A)DPV; (B) Response current histogram; (C) Five measurements of the same electrode in 0.1M CPB (pH=6.8).

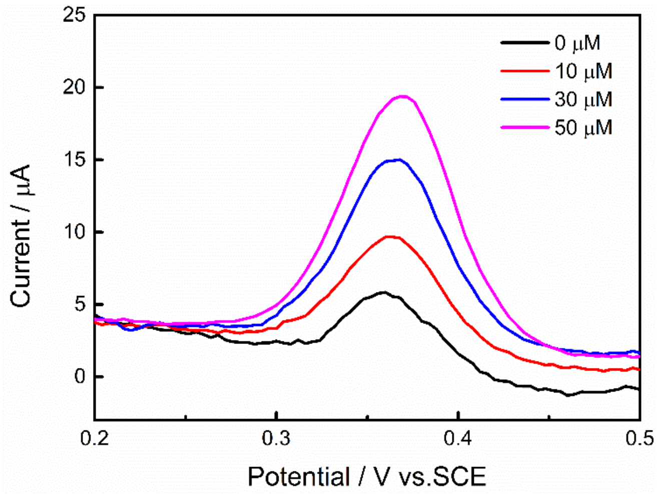

Fig S9. The DPV curve of paracetamol in the drug was determined in 0.1 MCPB (pH = 6.8).

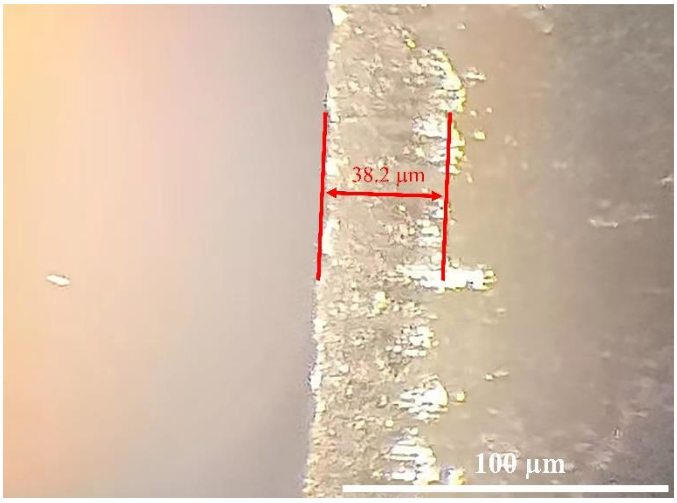

Fig S10 The thickness of the Fe-NC-800 layer deposited on GCE.

Table S1. C, N and Fe contents of Fe-NC group calculated from XPS results.

sample	C (wt%)	O(wt%)	N(wt%)	Fe (wt%)
Fe-NC-700	87.61	5.57	4.47	2.35
Fe-NC-800	88.61	5.33	4.02	2.04
Fe-NC-900	90.44	4.89	3.06	1.61

Table S2. Comparison of sensitivity and detection limits of electrodes modified with different materials

Modified electrodes	Sensitivity(μA μM^{-1} cm^{-2})	LOD(μM)
Bare GCE	0.767	0.129
NC-800/GCE	1.011	0.097
Fe-NC-700/GCE	1.908	0.052
Fe-NC-800/GCE	3.830	0.026
Fe-NC-900/GCE	2.188	0.046
